# Receptor-interacting protein kinase-3 (RIPK3): a new biomarker for necrotising enterocolitis in preterm infants

**DOI:** 10.1007/s00383-024-05697-x

**Published:** 2024-05-02

**Authors:** Lirong Shen, Zuming Yang, Chuchu Gao, Lili Li, Yu Wang, Yan Cai, Zongtai Feng

**Affiliations:** Department of Neonatology, The Affiliated Suzhou Hospital of Nanjing Medical University, Suzhou, Jiangsu Province, China

**Keywords:** Biomarker, Necrotising enterocolitis, Receptor-interacting protein kinase-3, Diagnostic value, Predictive value

## Abstract

**Objective:**

This study aimed to evaluate the role of receptor-interacting protein kinase-3 (*RIPK3*) in the diagnosis, estimation of disease severity, and prognosis of premature infants with necrotising enterocolitis (NEC).

**Methods:**

*RIPK3*, lactic acid (*LA*), and C-reactive protein (*CRP*) levels were measured in the peripheral blood of 108 premature infants between 2019 and 2023, including 24 with stage II NEC, 18 with stage III NEC and 66 controls. Diagnostic values of the indicators for NEC were evaluated via receiver operating characteristic (ROC) curve analysis.

**Results:**

Plasma *RIPK3* and *LA* levels upon NEC suspicion in neonates with stage III NEC were 32.37 ± 16.20 ng/mL. The ROC curve for the combination of *RIPK3, LA, CRP* for NEC diagnosis were 0.925. The time to full enteral feeding (FEFt) after recovery from NEC was different between two expression groups of plasma *RIPK3* (*RIPK3* < 20.06 ng/mL and *RIPK3* ≥ 20.06 ng/mL).

**Conclusion:**

Plasma *RIPK3* can be used as a promising marker for the diagnosis and estimation of disease severity of premature infants with NEC and for the guidance on proper feeding strategies after recovery from NEC.

## Introduction

In neonatal intensive care units, NEC is the leading cause of death due to gastrointestinal disease in premature infants, affecting 5–12% of neonates born at a very-low birth weight (VLBW) [1]. Nearly one-third of all cases are fatal, with a high risk of poor long-term growth and neurodevelopmental disorders in survivors [2]. Despite the study of NEC from various angles, the mechanisms that cause the disease are still mostly unknown. Therefore, it is necessary to develop effective strategies for the early diagnosis and estimation of disease severity of patients with NEC.

Necroptosis, which depends on a unique molecular pathway that does not overlap with apoptosis, is a type of programmed cell death that is mainly caused by tumour necrosis factor and toll-like receptor (*TLR*) family members, interferons, and other mediators [3], and its morphological features include cell swelling, mitochondrial dysfunction, and expanded cell membrane permeability. Necroptosis is associated with various pathological changes, such as photoreceptor extinction [4], systemic inflammation [5], distal colitis [6], and acute pancreatitis [7], and the common signalling pathway involved in necroptosis is the receptor-interacting protein kinase-3 (*RIPK3*)–*RIPK1*–mixed lineage kinase domain-like (MLKL) protein pathway [8]. RIPK1 is emerging as a master upstream regulator that controls cell survival and inflammatory signalling as well as multiple cell death pathways, including apoptosis and necroptosis. RIPK3 is a downstream mediator of RIPK1 in promoting necroptosis. RIPK3 phosphorylates MLKL, ultimately leading to the induction of necroptosis [9]. It is generally established that Toll-like receptors have a role in NEC pathogenesis. Necroptosis, which is activated in the intestinal epithelium upon TLR4 signalling, is necessary for the development of NEC [10, 11]. Therefore, we proposed a hypothesis that plasma *RIPK3* altered before the intestinal lesion was evident in NEC. Additionally, we hypothesized that infants with higher *RIPK3* levels would reach full enteral feeding longer than infants with lower *RIPK3* levels. Next, we measured the plasma levels of *RIPK3* and compared them with those of *LA* and *CRP* in preterm infants with NEC. The present study provides evidence for further exploration of the mechanism of necroptosis in NEC and its subsequent clinical applications.

## Materials and methods

All experiments involving humans complied with the relevant national regulations and institutional policies and were performed in accordance with the tenets of the Declaration of Helsinki. We performed a prospective study on premature infants with NEC hospitalized in the Department of Neonatology of the Affiliated Suzhou Hospital of Nanjing Medical University from September, 2019 to February, 2023. All experiments were conducted with informed consent of the legal guardians or parents of the patients. This study was reviewed and approved by the Medical Ethics Committee of the Affiliated Suzhou Hospital of Nanjing Medical University (approval number of ethical documents: K-2020-086-K01). Inclusion criteria were: (1) age < 28 d and (2) fulfilling the NEC criteria for preterm infants. NEC was diagnosed based on Bell’s diagnostic criteria [12]. Infants who met the following criteria were enrolled in the NEC group: (1) one or more systemic symptoms, including an unstable body temperature, drowsiness, bradycardia and apnoea, (2) one or more clinical signs, including gastric aspirate with emesis or bile, abdominal distension, and occult and/or gross bloody stools, and (3) at least one of the following imaging findings: portal vein gas, pneumatosis intestinalis, and/or pneumoperitoneum. Age- and gestational age-matched premature infants whose mothers had hypertensive or bleeding disorders were included as the controls. Exclusion criteria were: intestinal ischaemia–hypoxia secondary to perinatal asphyxia and congenital heart disease, isolated intestinal perforation, viral enteritis, enterocolitis associated with Hirschsprung’s disease, food protein-induced enterocolitis syndrome, immunodeficiency diseases, severe congenital malformations, inherited metabolic diseases, chromosomal anomalies, and lack of parental consent. According to Bell’s classification standard [13], infants were classified into suspected NEC (stage I), moderate NEC (stage II), and severe NEC (stage III) groups. In this study, infants with confirmed NEC represented those with stages II and III NEC.

**Fig. 1:**
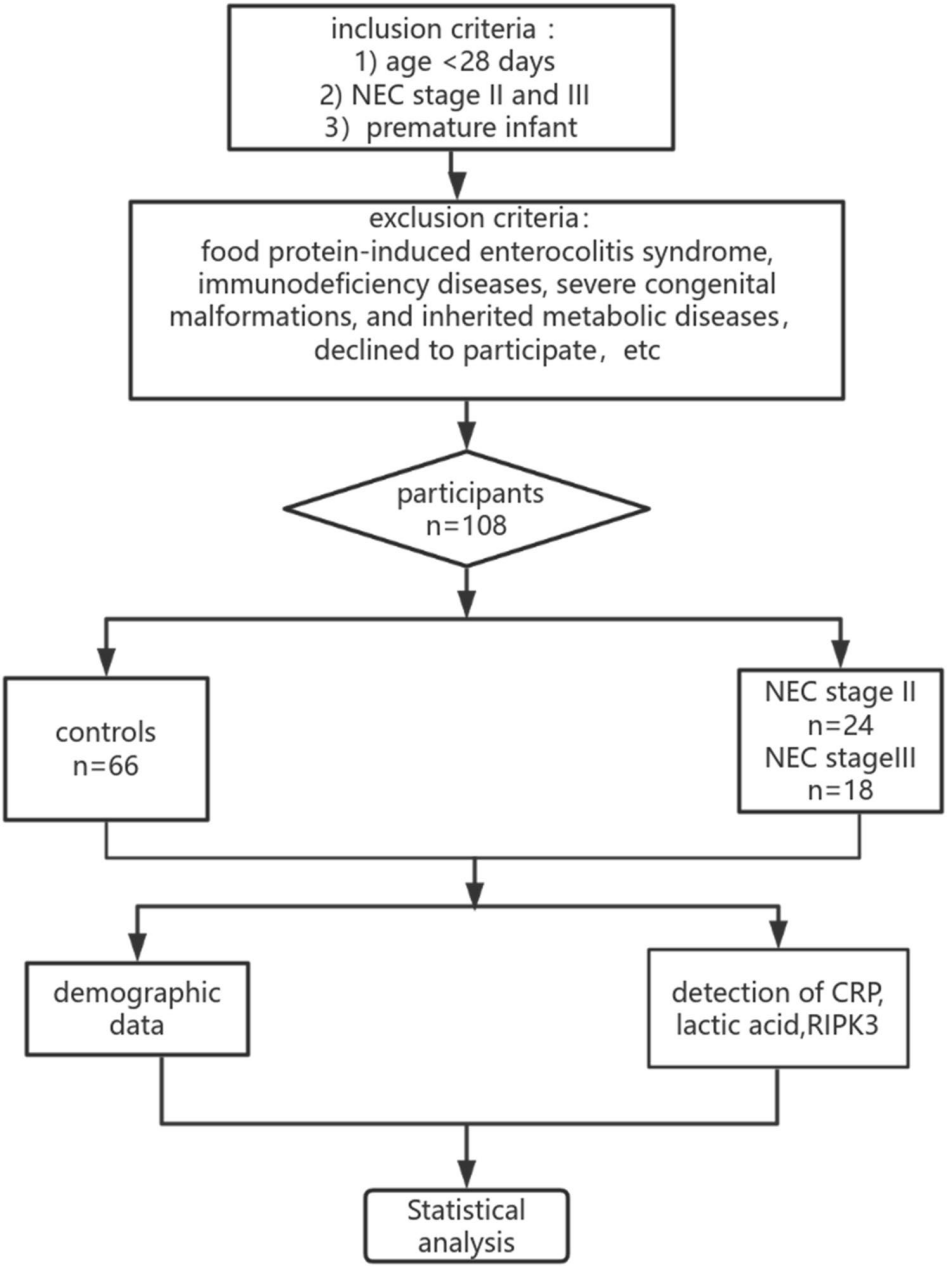
The flowchart of the proposed method. *RIPK3* receptor interacting protein kinase-3, *CRP* C-reactive protein. *NEC* necrotizing enterocolitis

The demographic and clinical characteristics of the premature infants were all recorded. Peripheral blood was collected upon NEC suspicion, 0.5 mL of which was administered in heparinised syringes and processed on a Radiometer ABL 80 analyser (Diamond Diagnostics, USA) for *LA*. Peripheral blood (1 mL) was collected in an ethylenediaminetetraacetic acid-containing tube for routine blood analysis and *CRP* was separated in the clinical laboratory via immune turbidimetry using an XE-2100 blood cell analyser (Sysmex Corporation, Kobe, Japan) and centrifuged (1610 × *g*, 10 min). The supernatant filtrate was collected and stored at –80 °C for the detection of *RIPK3*. We measured *RIPK3* levels in the plasma using enzyme-linked immunosorbent assay (ELISA) kits (CUSABIO Technology LLC, Wuhan, China). In controls, *RIPK3*, *LA,* and *CRP* levels were determined using the same methodology. A flow diagram depicting the study process is shown in Fig. 1.

### Statistical analysis

Statistical Package for the Social Sciences (SPSS) version 20.0 (IBM Corporation, Armonk, NY, US) was used to evaluate the relationships among the variables. The normality of the distribution of quantitative data was determined using the Kolmogorov–Smirnov test. Data with a normal distribution, represented as the mean ± standard deviation, were compared using analysis of variance or student’s *t* test. Quantitative data with a non-normal distribution are represented as the median and interquartile range and compared using the nonparametric rank sum test and Kruskal–Wallis Test. Categorical data are presented as numbers (percentages) and compared using the chi-square test or Fisher’s exact test. To determine the optimal cut-off values of plasma *RIPK3*, LA, and *CRP* for the prediction of NEC, receiver operating characteristic (ROC) curve analysis was performed using IBM SPSS Statistics version 20. NEC (stages II and III) and the controls showed state variable values 1 and 0, respectively, in the ROC curve with a *p*-value < 0.05, confirming the statistical significance of the model. Differences with a *p*-value < 0.05 were considered to be statistically significant.

## Results

### Study population and baseline information

A total of 108 premature infants were enrolled in this study: 42 in the NEC groups (24 in NEC II and 18 in NEC III groups) and 66 in the control group. The mean birth weight and mean gestational age were 1398.33 ± 316.97 g and 30.35 ± 1.81 weeks, respectively, in NEC II group and 1437.37 ± 391.71 g and 30.46 ± 2.00 weeks, respectively, in NEC III group. The mean gestational age and birth weight were 30.63 ± 2.11 weeks and 1543.95 ± 369.36 g in the controls. There were no significant differences between cases and controls in gestational age, birth weight, sex, age at sampling, Apgar score, amniotic fluid status, small for gestational age, gestational hypertension, antenatal glucocorticoids, premature rupture of membranes, mode of delivery, and time of initiation of feeding. Compared with NEC II group, NEC III group had lower platelet count at clinical onset, higher incidences of septicemia, shock, respiratory failure, surgery, peritonitis, and longer time to achieve full intestinal feeding after NEC as well as longer hospitalization stays (*P* < 0.05). No significant differences in other factors, including general information, prenatal risk factors and other risk factors were observed (*P* > 0.05) (Table 1). Demographic data are summarised in Table 1.

**Table 1:** Clinical features of the infants enrolled in this study

Variables	NEC II *n* = 24	NEC III *n* = 18	Control *n* = 66	*χ*^2^/*Z*/*t*	*P*-value
Gestation age (weeks), mean ± SD	30.35 ± 1.81	30.46 ± 2.00	30.63 ± 2.11	3.58^a^	0.09
Birth weight (g), mean ± SD	1398.33 ± 316.97	1437.37 ± 391.71	1543.95 ± 369.36	1.42^a^	0.24
Male (%)	13 (58)	12 (68)	42 (65)	0.51^b^	0.77
Age at samples taken, (days),mean ± SD	14.83 ± 5.57	14.37 ± 8.46	11.40 ± 6.92	2.34^a^	0.10
1 min Apgar score	4.61 ± 1.22	4.41 ± 0.94	5.06 ± 1.33	0.72^a^	0.48
5 min Apgar score	7.12 ± 1.61	5.91 ± 2.35	7.37 ± 1.56	2.01^a^	0.14
amniotic fluid condition (clear)	23 (96)	18 (100)	64 (98)	0.11^b^	0.71
Small for gestational age (%)	5 (22)	4 (24)	17 (26)	0.12^b^	0.73
Hypertension of pregnancy (%)	7 (30)	5 (29)	19 (30)	0.00^b^	1.00
Glucocorticoids prenatally (%)	17 (72)	13 (74)	52 (80)	0.55^b^	0.45
Premature rupture membranes (%)	6 (27)	4 (25)	25 (39)	0.89^b^	0.34
Natural labour	9 (40)	9 (50)	28 (43)	0.35^b^	0.55
Feeding start time	2.00 (1.00, 3.00)	2.00 (2.00, 3.00)	2.00 (1.00, 3.00)	− 0.53^c^	0.61
Time to achieve full intestinal feeding after NEC	8.00 (6.00, 10.25)	20.5 (15.5, 39.75)	/	− 2.68^c^	0.00
NEC diagnosis time	16.00 (11.00, 27.00)	22.5 (16.25, 27.87)	/	− 1.54^c^	0.12
Antibiotic use before onset	8 (36)	7 (41)	/	0.09^b^	0.76
WBC at clinical onset	7.92 (6.37, 9.86)	7.17 (5.18, 12.01)	/	− 0.25^c^	0.81
PLT at clinical onset	278.80 ± 20.92	225.8 ± 25.99	/	1.43^a^	0.01
CRP at clinical onset	0.50 (0.50, 2.89)	0.72 (0.50, 8.40)	/	− 0.86^c^	0.43
Respiratory failure (%)	6 (26)	18 (100)	/	18.48^b^	0.00
Intestinal perforation(%)	0 (0)	1 (8)	/	/^d^	0.28
Peritonitis (%)	1 (3)	16 (91)	/	28.58^b^	0.00
Shock (%)	4 (20)	18 (100)	/	22.40^b^	0.00
Septicemia (%)	9 (40)	18 (100)	/	12.6^b^	0.00
Mortality (%)	0 (0)	2 (16)	/	/^d^	0.07
Hospital stay (%)	40.97 ± 3.96	57.25 ± 6.77	/	− 2.14^a^	0.03
Surgery (%)	2 (10)	14 (81)	/	16.73^b^	0.00
Death (%)	0 (0)	3 (16)	/	/^d^	0.07
Formula milk feeding (%)	24 (100)	18 (100)	66 (100)	/	/

Data are presented as the mean ± standard deviation, median and interquartile range or count. *NEC* necrotising enterocolitis

^a^Analysis of variance

^b^*χ*^2^ test

^c^Mann-Whitney test

^d^Fisher’s exact test

### Comparisons of plasma levels of RIPK3, LA, and CRP in the three groups

The level of RIPK3 in each group was 16.51 ± 7.46, 25.62 ± 4.03, and 32.37 ± 16.20 ng/mL respectively, the level of LA in each group was 1.00 (0.80, 1.20), 1.30 (0.80, 1.80), and 2.10 (1.27, 6.00) mmol/L, respectively, and the level of CRP in each group was 4.50 (1.75, 13.00), 12.00 (3.00, 16.50), and 30.50 (10.62, 38.00) mg/L, respectively. The differences were significant among the three groups (*P* < 0.05) (Table 2).

**Table 2:** Comparison of levels of plasma *RIPK3, lactic acid* and *CRP* between the control and patients with NEC

Variables	RIPK3 (ng/mL)	LA (mmol/L)	CRP (mg/L)
Control (*n* = 66)	16.51 ± 7.46	1.00 (0.80, 1.20)	4.50 (1.75, 13.00)
NEC II (*n* = 24)	25.62 ± 4.03	1.30 (0.80, 1.80)	12.00 (3.00, 16.50)
NEC III (*n* = 18)	32.37 ± 16.20	2.10 (1.27, 6.00)	30.50 (10.62, 38.00)
F/H	− 45.92^a^	30.33^b^	11.08^b^
*P*-value	0.000	0.000	0.004

Data are presented as the mean ± standard deviation, median and interquartile range. *NEC* necrotising enterocolitis, *RIPK3* Receptor-interacting protein kinase-3, *LA* lactic acid, *CRP* C-reactive protein

^a^One-Way ANOVA

^b^Kruskal-Wallis test

### ROC curve analysis of the predictive values of RIPK3, LA, and CRP levels

**Fig. 2:**
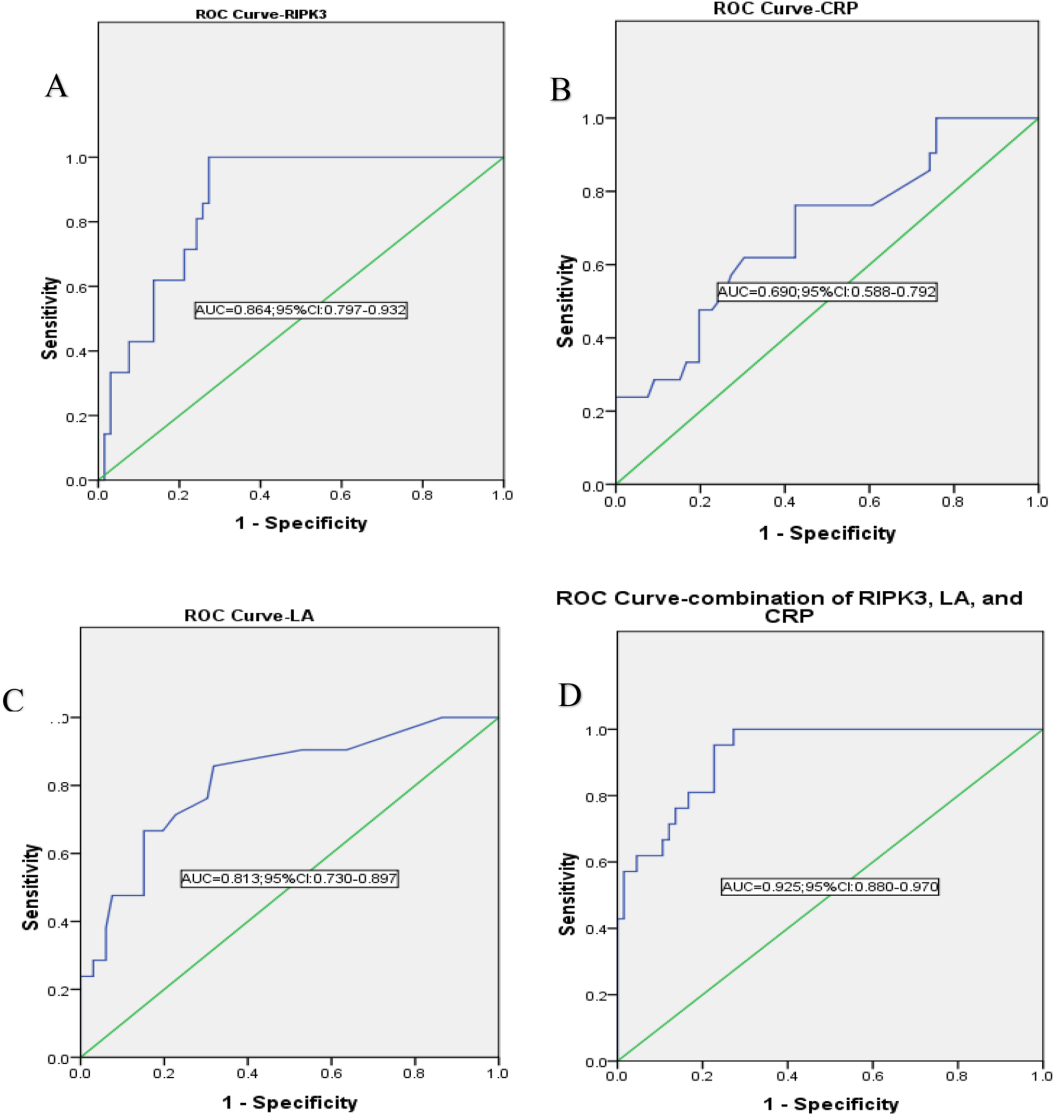
ROC curve for the ability of *RIPK3* (**A**), *CRP* (**B**), *LA* (**C**) and combination of *RIPK3, LA* and *CRP* (**D**) to predict NEC. *AUC* area under the receiver operating characteristic curve, *CI* confidence interval, *RIPK3* receptor interacting protein kinase-3, *LA* lactic acid, *CRP* C-reactive protein

The sensitivity, specificity, and cut-off values for these parameters were determined using the ROC curve (Fig. 2). *RIPK3* had a cut-off point of 20.52 ng/mL, with 100% sensitivity, 72.7% specificity, and 0.72 Youden index with area under the receiver operating characteristic curve (AUC) of 0.864 (Fig. 2A). *CRP* had a cut-off point of 6.25 mg/L, with 76.2% sensitivity, 57.6% specificity, and 0.33 Youden index with AUC of 0.690 (Fig. 2B). *LA* had a cut-off point of 1.05 mmol/L, with 85.7% sensitivity, 68.2% specificity, and 0.53 Youden index with AUC of 0.813 (Fig. 2C). ROC curve plotted to examine the performance of the combination of *RIPK3, LA,* and *CRP* had an AUC of 0.925, which was higher than that for any single index (Fig. 2D).

### Effect of plasma RIPK3 levels on the recovery of FEFt

**Fig. 3:**
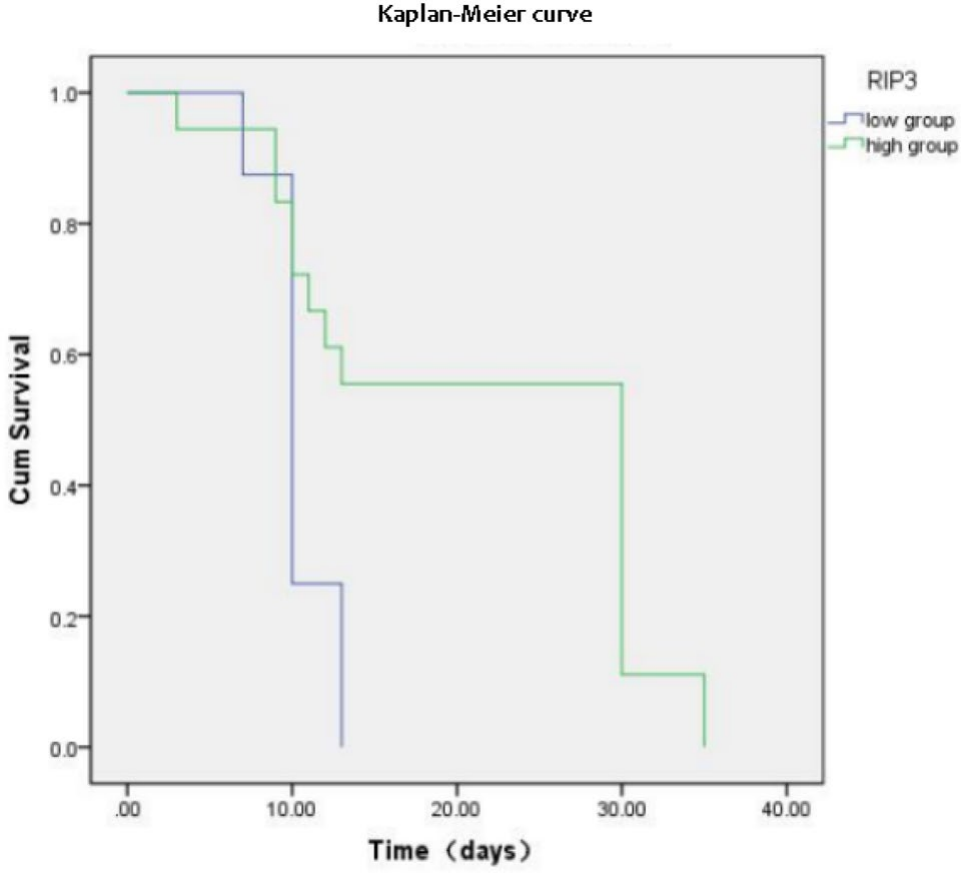
Kaplan–Meier diagram showing results on the time to full enteral nutrition. *RIPK3* receptor interacting protein kinase-3

Preterm infants with NEC were divided into the low-expression group (*RIPK3* < 20.06 ng/mL) and high-expression group (*RIPK3* ≥ 20.06 ng/mL) according to the 75th percentile of plasma *RIPK3* levels in the survival group. Kaplan–Meier survival analysis revealed that high *RIPK3* levels were associated with a long recovery FEFt after premature recovery from NEC (*p* = 0.006) (Fig. 3). Kaplan–Meier curves for FEFt between the two groups are shown in Fig. 3. The median FEFt was 10 d (95% confidence interval [CI] = 8.56–11.44) in the low-expression group and 30 d (95% CI = 26.06–33.92) in the high-expression group.

## Discussion

*RIPK3* is an essential protein in the “programmed” and “regulated” cell death pathway called necroptosis [14]. A large proportion of current studies focus on the underlying molecular mechanisms of necroptosis in the pathophysiological processes of diseases [15–18]. However, few studies have been conducted to investigate the clinical application of *RIPK3* as a biomarker. To the best of our knowledge, the present prospective study is the first to evaluate the predictive value of plasma *RIPK3* as a biomarker for the diagnosis, severity and prognosis of NEC.

We found that plasma *RIPK3* at onset in neonates with stage III NEC were higher than those in neonates with stage II NEC. Plasma *RIPK3, LA and CRP* levels in neonates with NEC were significantly higher than those in the controls. We also compared the average age of the babies when samples were taken and the days of NEC diagnosis, and found that there was significant difference between two groups (*t* = 3.55, *P* = 0.001), which helped to determine that *RIPK3* is effective in predicting NEC onset. These results suggested that plasma *RIPK3* expression may be associated with the diagnosis and severity of NEC, which facilitates neonatologists to timely and confidently reach a decision for early transfer of infants with NEC to tertiary surgical centers. Many biomarkers of NEC have been reported in the serum, stool, and urine [19]. However, their clinical application is limited due to various reasons, especially their low specificity [20]. Some intestinal markers, such as plasma intestinal fatty acid-binding protein (*I-FABP*), are promising biomarkers that exhibit high specificity for the diagnosis of NEC, but their use is restricted due to their moderate sensitivity and lack of appropriate identification facilities in neonatal intensive care units [21]. Although the specificity of *RIPK3* is lower than that of plasma *I-FABP*, according to two meta-analyses reports [21, 22], it can be detected in the blood volume from routine blood tests with very high sensitivity at onset. Moreover, the areas under the ROC curve for the combination of *RIPK3, LA* and *CRP* for NEC diagnosis were higher than the single indicator, and integration of *RIPK3*, *LA*, and *CRP* levels aids in the robust diagnosis of NEC.

Zhang [23] found that circulating RIPK3 was significantly increased and correlated positively with markers of enterocyte injury in critically ill patients with intestinal injury. Intestinal RIPK3 and MLKL were phosphorylated after 2 h of intestinal reperfusion. These findings are consistent with the results of our study. Although apoptosis has long been recognized as the major mode of intestinal epithelial cell (IEC) death in NEC [24]. Apoptosis, as a non-inflammatory type of cell death, does not appear to be sufficient to explain the rapid and intense inflammatory response in NEC. Apoptosis mainly occurs in intestinal crypt epithelial cells (e.g., Paneth cells) [25], whereas necroptosis mainly occurs in intestinal epithelial villus cells [11]. Differences in the types of death of IECs may be related to their different biological roles in the pathogenesis of NEC. Apoptosis of intestinal stem cells can lead to a disruption of intestinal mucosal epithelial repair, impairing the restoration of intestinal epithelial barrier (IEB) function [26]. Necroptosis explains the rupture of intestinal villi in NEC, which facilitates microbial translocation across the IEB and promotes the development of late onset sepsis.

Studies confirmed the role of necroptosis in both NEC models and human surgical specimens [11, 12, 27]. Most samples used for *RIPK3* detection have been from animal or human tissues. To date, only two studies have tested *RIPK3* levels in human blood, one [28] in patients with septicaemia and the other [29] in occupational Al-exposed workers. In the two studies, *RIPK3* levels detected via ELISA in the case group were higher than those in the controls. In our study, plasma *RIPK3* in neonates with stage III NEC were higher than those in stage II NEC, but the difference of *CRP* between the two group was not statistically significant, which may indicate that necroptosis is the cause of inflammation indirectly.

Prolonged FEFt after NEC in preterm-born children surviving NEC is associated with lower motor composite and cognitive scores at the age of 2–3 years [30]. It is difficult to predict intestinal recovery after NEC in individuals. Feenstra FA [31] found that levels of plasma citrulline at 0–24 h were higher in infants with FEFt ≤ 20 days than in infants with FEFt > 20 days. We found that it took longer FEFt in the high-expression group (*RIPK3* ≥ 20.06 ng/mL). This result indicates that *RIPK3* can aid in the guidance to optimize and individualize NEC treatment in preterm infants. Further evaluation of *RIPK3* can provide new insights into the pathogenesis of NEC. Moreover, necroptosis inhibitors may alleviate NEC injury, and we aim to investigate this in future studies.

## Limitations

We are aware of some limitations. First, the volume of blood collected was relatively small to prevent anaemia, making it difficult to detect additional indicators of NEC. Second, there was a lack of monitoring at multiple time points to explore the dynamic expression of different NEC indicators. Third, it was difficult to obtain blood samples from premature infants under physiological conditions; hence, the sample size was small. Moreover, we could not compare the expression levels of *RIPK3* and other biomarkers, such as MLKL faecal calprotectin, *I-FABP*, claudins, and trefoil factor 3, between different gestational ages and body weights under physiological conditions for the prediction and diagnosis of NEC. Fourth, due to the limited number of NEC stage III cases, it was not possible to extensively investigate NEC with different aetiologies. Fifth, when surgery is required, patients are transferred to an affiliated tertiary hospital from which we can’t easily obtain surgical specimens. Therefore, plasma RIPK3 levels with histological findings related to the necroptosis-apoptosis cross-talk in surgical patients are difficult to obtain. Lastly, this was a single-centre preliminary study with a small sample size. Therefore, our findings need to be confirmed by further large-scale, long-term, and multicentre studies.

## Conclusion

In this study, we found that plasma *RIPK3* was a promising biomarker for the early diagnosis of NEC and estimation of disease severity. We also observed plasma *RIPK3* was helpful in the guidance on proper feeding strategies after recovery from NEC.

## Data Availability

The datasets used and/or analyzed during the current study are available from the corresponding author on reasonable request.

## Abbreviations

NEC: Necrotising enterocolitis
*RIPK3*: Receptor-interacting protein kinase-3
LA: Lactic acid
CRP: C-reactive protein
FEFt: Time to full enteral feeding
ROC: Receiver operating characteristic
VLBW: Very-low birth weight
TLR: Toll-like receptor
ELISA: Enzyme-linked immunosorbent assay
SPSS: Statistical Package for the Social Sciences
AUC: Area under the receiver operating characteristic curve
CI: Confidence interval
I-FABP: Intestinal fatty acid-binding protein
